# The influence of demographic and lifestyle factors on blood donation delay among student population: a retrospective study

**DOI:** 10.3389/fpubh.2023.1297472

**Published:** 2023-12-06

**Authors:** Wang Feng, Wang Yun, Wang Le, Xu Zhi-guo, Yang Hai-ying, Wu Shu-fang, Wei Zhen-yan, Chen Yi-zhu, Sun Quan, Fei Jing-xian

**Affiliations:** ^1^Huzhou Central Blood Station, Huzhou, Zhejiang, China; ^2^Huzhou College, Huzhou, China

**Keywords:** donor deferral, characteristic analysis, students, sleep quality, dietary situation, stress

## Abstract

**Objective:**

This study analysed blood donation deferral trends, reasons and demographic/lifestyle characteristics among students in Huzhou City. The aim was to understand the health status of students and reduce the deferral rate.

**Methods:**

Data on blood donation deferral among students in Huzhou City from 2018 to 2022 were collected and analysed. Deferral trends and main reasons were investigated. Using demographic and lifestyle data from 2,619 cases in 2022, a risk prediction model for deferral was constructed.

**Results:**

The deferral rate among students in Huzhou City from 2018 to 2022 was 12.60% (*p* = 0.000, 95%CI: 12.14–13.06%), showing a significant increasing trend. Temporary deferral was the main reason, with alanine aminotransferase (ALT), blood pressure (BP) and haemoglobin (Hb) as the main deferral factors. ALT had a deferral rate of 5.23% (4.92–5.53%), BP 3.30% (3.06–3.55%), and Hb 2.92% (2.68–3.15%). Demographic and lifestyle characteristics in 2022 showed no significant differences between education level, household registration and deferral rate (*p* > 0.05). However, age, sex, blood donation history, sleep quality, diet and mental state had variable effects on ALT, BP, and Hb deferrals (*p* < 0.05). Logistic regression showed that sex, blood donation history, sleep quality, diet and mental status were independent risk factors for ALT deferral (*p* < 0.05), with odds ratios (ORs) of 5.057, 2.735, 1.594, 3.679, and 1.957, respectively. Age, blood donation history, sleep quality and mental state were independent risk factors for BP deferral (*p* < 0.05), with ORs of 0.256, 3.658, 6.042, and 1.812, respectively. Gender, blood donation history and diet were independent risk factors for Hb deferral (*p* < 0.05), with ORs of 0.244, 0.542, and 3.103, respectively.

**Conclusion:**

Students’ health problems require attention. Effective health education should improve self-health management and pre-donation health behaviour to encourage regular blood donation.

## 1 Introduction

The blood donor screening process is critical to ensuring blood safety. The recruitment, selection, and retention of voluntary, unpaid blood donors from low-risk populations is the cornerstone of ensuring a safe, sustainable, and adequate blood supply. Against the backdrop of an ageing global population, the growing demand for blood has become the norm. Research predicts that changes in China’s population structure will result in a 16.0% decrease in blood supply between 2016 and 2036. To maintain a balance between blood supply and demand, annual blood supply growth of 0.9 to 1.8% is required, but this growth may vary due to regional differences (1). Therefore, how to better develop and maintain a pool of blood donors is a pressing issue for countries around the world.

During the donation process, standardised screening of donors is a common practice worldwide to reduce unnecessary waste of blood resources. However, pre-donation screening may lead to temporary or permanent deferral of some donors. Previous studies have shown that deferral has a negative impact on future donation behaviour, suggesting that deferred donors are less likely to return for subsequent donations. This effect is particularly pronounced among first-time donors, who are more sensitive to deferral, and is detrimental to the development of a regular donor pool (2–6). It is therefore essential to address the health issues of eligible donors, especially young students who are the future mainstay of blood donation. Finding ways to reduce deferral rates is a pressing issue. A timely understanding of the changing patterns of blood donation deferral in this specific population is critical for countries undergoing significant demographic changes to ensure the sustainability and safety of the blood supply.

## 2 Methods

### 2.1 Design and participants

From 2018 to 2022, a total of 20,132 students participated in voluntary blood donation activities in Huzhou City. The number of participants in each year was 4,716, 4,239, 3,755, 4,783, and 2,619, respectively. The health assessment and pre-donation testing of blood donors are conducted by qualified evaluators in accordance with China’s “Blood Donor Health Examination Standards.” Donors undergo various tests before donating blood, including physical examinations such as blood pressure, pulse, weight, general condition, and blood tests for haemoglobin (Hb), alanine aminotransferase (ALT), hepatitis B surface antigen (HBsAg), and syphilis antibody (anti-TP). Hb, blood lipids and ALT are tested using a semi-automated biochemical analyser, while HBsAg and anti-TP are tested using a combination of rapid test strips. Prospective donors who do not meet the requirements of the physical examination or blood tests are temporarily or permanently deferred from donating blood. Temporary deferrals include problems found during health screening, such as failure to meet weight standards, abnormal blood pressure, and elevated ALT, low haemoglobin levels, and chylous blood found during pre-donation testing. Chylous blood refers to the presence of chyle mixed in the blood, characterized by a turbid milky appearance with fat droplets, cell debris, and chyle particles. Although the “Health Examination Standards for Blood Donors” in China does not include this specific item, individuals with chylous blood are typically deferred from blood donation due to concerns regarding the accuracy of testing results and potential harm to recipients during transfusion. Permanent deferrals include serious illness, high-risk behaviour and positive hepatitis B or syphilis tests. As these reasons are less common in the student population, they will be classified as “permanent deferrals” for statistical analysis. Estimate and compare the deferral rates and 95% confidence intervals (CI) of student blood donors for the years 2018–2022, and determine if there are significant differences in deferral rates between each year. In addition, analyse and compare the trends in the main deferral categories over the five-year period using the Pareto principle. This study received ethical approval from the Ethics Committee of Huzhou Central Blood Station on 16 November 2021 (approval number: IRB20211116). Before formally agreeing to participate in the study, all participants underwent a detailed informed consent procedure. They were informed of the purpose of the study, the expected outcomes, the potential risks and benefits, and that they could withdraw from the study at any time. All personal identifying information and other sensitive data of the participants were treated with strict confidentiality. Data were stored anonymously and were only accessible to the researchers to ensure data protection and confidentiality.

### 2.2 Survey and demographic data collection

We collected the living characteristics and demographic data of 2,619 students who donated blood in 2022 through telephone follow-up and database queries. We excluded donors with unsuccessful follow-up or incomplete database information, resulting in a total of 2,321 complete data entries. These included 147 individuals with delayed ALT (alanine aminotransferase), 108 individuals with delayed BP (blood pressure) and 131 individuals with delayed Hb (haemoglobin). These 386 delayed blood donors were assigned to the research group and named “ALT Delayed Group,” “BP Delayed Group” and “Hb Delayed Group” respectively. The data of 1,935 students who successfully donated blood were collected as the “control group.” Life characteristic data were obtained by telephone follow-up and included three aspects: sleep assessment using the Jenkins Sleep Evaluation Questionnaire (7), which asked about the frequency of the following symptoms in the previous 4 weeks: (1) difficulty falling asleep, (2) waking up several times during the night, (3) difficulty staying asleep, and (4) feeling tired and exhausted on waking after the usual sleep time. Each question had a 6-point response scale, and the overall sleep quality score was used for classification. A total score of 8 or less indicated better sleep quality, while a score above 8 indicated worse sleep quality. Perceived stress was assessed using the Chinese Perceived Stress Scale (CPSS) (8), which measures an individual’s subjective perception of stress. It consisted of 14 items with a Likert scale ranging from 0 to 4. A higher score indicated a higher perceived psychological stress level, and a CPSS score of 26 or higher indicated health risks due to stress. The dietary assessment criteria included six questions that measured students’ eating habits, including eating speed, regular and quantified meals, non-picky eating, avoidance of late night snacks, balanced diet, and limited consumption of snacks. Each question had a 5-point response scale, with a total score of 8 or less indicating better dietary status and a score above 8 indicating poorer dietary status. Demographic data included age, sex, education level, place of household registration and blood donation history, which were obtained from the Blood Information Management System (BIS), which we used for data retrieval and analysis. Regarding the classification of blood donation frequency, according to the World Health Organization (WHO) definition, regular blood donors referred to voluntary, unpaid blood donors who had donated blood at least three times, with the last donation within the previous year and at least one regular donation per year. Repeat donors were individuals who had previously donated blood, but with an interval of more than 1 year between two donations (9). Considering that the proportion of regular blood donors among students is extremely low, we categorised blood donation history into two groups: “repeat donors” and “first-time donors.” The aim was to analyse and compare the differences in life history data between the delayed group and the control group, as well as the differences in delay rates between different demographic characteristics of blood donors.

### 2.3 Statistical analysis

To compare lifestyle and demographic characteristics, variables with significant differences were selected as independent variables and the presence of delay was used as the dependent variable. Logistic regression analysis was used to further analyse and identify the main causes and risk factors for blood donation delay. Data were entered into an Excel spreadsheet and analysed using SPSS version 23 software from IBM, Armonk, New York. GraphPad Prism 9.0 software was used for graphing, and categorical variables were expressed as frequencies and percentages. Chi-squared test was used to compare proportions, t-tests or Mann–Whitney U test were used to compare means, and logistic regression analysis was used to build a risk assessment model and calculate odds ratios and their respective 95% confidence intervals for each factor. All statistical tests were two-tailed, and a significance level of *p* < 0.05 was considered statistically significant.

## 3 Results

### 3.1 Distribution of reasons for delayed blood donation

From 2018 to 2022, a total of 20,132 students participated in blood donation. Among them, there were 2,536 cases of delayed blood donation, resulting in a delay rate of 12.60% (95% CI, 12.14–13.06%). Looking at the reasons for delay, the temporary delay rate caused by factors such as blood pressure (BP), haemoglobin (Hb), and alanine aminotransferase (ALT) was significantly higher than the permanent delay rate caused by hepatitis B, syphilis, severe illness and other factors. Specifically, the temporary delay rate was 12.31% (95% CI, 11.86–12.77%), while the permanent delay rate was only 0.28% (95% CI, 0.21–0.36%). Among all delayed students, temporary delays accounted for 97.75% of cases. Among these, ALT, BP, and Hb were the main causes of delay. These three factors together accounted for 90.85% of all delays. In order of delay rates, they were 5.23% (95% CI, 4.92–5.53%), 3.30% (95% CI, 3.06–3.55%), and 2.92% (95% CI, 2.68–3.15%), respectively. The delay proportions for each factor are shown in Table 1.

**Table 1:** Classification of reasons for delayed blood donation in the student population (*n* = 20,132).

projects	Number of delayed blood donations(*n*)	Deferral rate/100 (95%CI)
Temporary Delay	2,479	12.31 (11.86–12.77)
Weight	137	0.68 (0.57–0.79)
BP	665	3.30 (3.06–3.55)
Hb	587	2.92 (2.68–3.15)
ALT	1,052	5.23 (4.92–5.53)
Coeliac blood	38	0.19 (0.13–0.25)
Permanent delay	57	0.28 (0.21–0.36)

### 3.2 Trends in delayed blood donation rates

After 5 years of data analysis, it can be observed that the rate of blood donation delays is gradually increasing. The specific data are as follows: In 2018, there were 4,716 participants, with 442 instances of delayed donations and a delay rate of 9.37% (8.54–10.20%). In 2019, there were 4,239 participants with 459 cases of delayed donations and a delay rate of 10.83% (9.89–11.76%). In 2020, there were 3,755 participants with 491 cases of delayed donations and a delay rate of 13.08% (12–14.15%). In 2021, there were 4,783 participants with 653 cases of delayed donations and a delay rate of 13.65% (12.68–14.63%). In 2022, there were 2,619 participants with 491 cases of delayed donation and a delay rate of 18.75% (17.25–20.24%).

**Figure 1:**
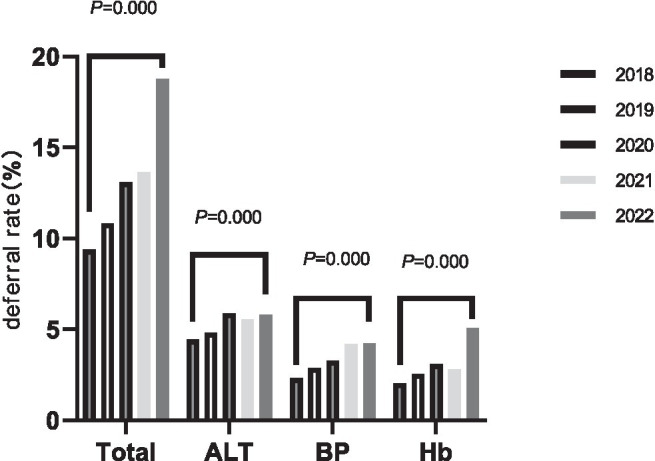
Trends in delayed blood donation rates in the student population, 2018–2022.

Looking at the data over the five-year period, the delay rate increases each year and the differences are statistically significant (*χ*^2^ = 152.066, *p* = 0.000). For the three main factors causing delays (ALT, BP, and Hb), their delay rate changes from 2018 to 2022 are as follows:

ALT delay rates were 4.43% (3.84–5.02%), 4.81% (4.17–5.46%), 5.89% (5.13–6.64%), 5.56% (4.91–6.21%), and 5.80% (4.91–6.70%), *χ*^2^ = 13.607, *p* = 0.009. BP delay rates were 2.31% (1.88–2.74%), 2.88% (2.37–3.38%), 3.28% (2.71–3.85%), 4.18% (3.61–4.75%), and 4.24% (3.47–5.01%), *χ*^2^ = 35.620, *p* = 0.000. The Hb delay rates were 2.04% (1.63–2.44%), 2.55% (2.08–3.03%), 3.09% (2.54–3.64%), 2.80% (2.33–3.27%), and 5.08% (4.24–5.92%), *χ*^2^ = 58.682, *p* = 0.000. See Figure 1 for more details.

### 3.3 Comparison of delay rates across demographics and life characteristics

In the three major retardation projects, the differences in demographic and lifestyle characteristics between the retardation group and the control group vary. In the ALT project, the differences in age, education level, and place of residence are not significant, but there are significant differences in five factors, including gender, blood donation history, sleep status, dietary status, and mental status. The differences are as follows: female vs. male, 0.98% (0.40–1.55%) vs. 14.23% (12.01–16.44%), *p* = 0.000; repeat vs. first time donors, 5.07% (3.16–6.97%) vs. 7.71% (6.39–9.03%), *p* = 0.042; good vs. poor sleep quality, 5. 54% (4.50–6.59%) vs. 18.60% (13.66–23.53%), *p* = 0.000; good nutritional status vs. poor nutritional status, 5.32% (4.15–6.50%) vs. 10.70% (8.36–13.04%), *p* = 0.000; good mental status vs. poor mental status, 6.07% (4.83–7.32%) vs. 9.16% (6.96–11.36%), *p* = 0.010. In the BP project, the differences in gender, education level, place of residence and nutritional status are not significant, while there are significant differences in four factors, including age, blood donation history, sleep status and mental status. The differences are as follows: age 18–20 vs. 21+ years, 6.46% (4.92–8.01%) vs. 4.21% (3.01–5.42%), *p* = 0.023; repeat vs. first time donors, 3.36% (1.17–5.04%) vs. 5.82% (4.67%-6. 97%), *p* = 0.040; good sleep quality vs. poor sleep quality, 4.2% (3.27–5.13%) vs. 12.84% (8.72–16.96%), *p* = 0.000; good mental state vs. poor mental state, 4.34% (3.27–5.40%) vs. 7.39% (5.35–9.43%), *p* = 0.004. In the Hb project, the differences in education level, place of residence, sleep quality and mental condition are not significant, while there are significant differences in four factors including age, sex, blood donation history and nutritional status. The differences are as follows: age 18–20 vs. 21+ years, 7.03% (5.72–8.33%) vs. 4.61% (2.91–6.31%), *p* = 0.042; female vs. male, 9.84% (8.13–11.55%) vs. 1.78% (0.92–2.65%), *p* = 0.000; repeat vs. first time donors, 3.97% (2.38–5.57%) vs. 7.26% (5.94–8.58%), *p* = 0.006; good vs. poor nutritional status, 4.36% (3.27–5.45%) vs. 10.08% (7.87–12.30%), *p* = 0.000. See Table 2 for detailed data.

**Table 2:** Comparison of delayed blood donation rates among student populations with different demographic and life characteristics.

Category	ALT, *n* = 2,082 (147)		PB, *n* = 2,043 (108)		Hb, *n* = 2,066 (131)	
	Prevalence per 100 (95% CI)	*p*	Prevalence per 100 (95% CI)	*p*	Prevalence per 100 (95% CI)	*p*
Age		0.476		0.023		0.042
18–20	7.40 (6.01–8.80)		6.46 (4.92–8.01)		7.03 (5.72–8.33)	
21+	6.55 (4.72–8.39)		4.21 (3.01–5.42)		4.61 (2.91–6.31)	
Sex		0.000		0.181		0.000
Female	0.98 (0.40–1.55)		5.82 (4.51–7.13)		9.84 (8.13–11.55)	
Male	14.23 (12.01–16.44)		4.47 (3.04–5.90)		1.78 (0.92–2.65)	
Education		0.094		0.592		0.138
Bachelor or above	6.32 (4.98–7.65)		5.51 (4.22–6.80)		5.71 (4.42–6.99)	
Below Bachelor	8.25 (6.34–10.16)		4.97 (3.50–6.44)		7.34 (5.53–9.14)	
Birthplace		0.663		0.652		0.116
Local	6.74 (4.95–8.53)		5.56 (3.98–7.15)		7.35 (5.59–9.12)	
Overseas	7.25 (5.85–8.64)		5.11 (3.88–6.34)		5.64 (4.35–6.94)	
Donor history		0.042		0.040		0.006
Repeat	5.07 (3.16–6.97)		3.36 (1.17–5.04)		3.97 (2.38–5.57)	
First time	7.71 (6.39–9.03)		5.82(4.67–6.97)		7.26 (5.94–8.58)	
Sleep Quality		0.000		0.000		0.143
Good	5.54 (4.50–6.59)		4.2 (3.27–5.13)		6.05 (4.95–7.15)	
Poor	18.60 (13.66–23.53)		12.84 (8.72–16.96)		8.47 (4.98–11.96)	
dietary situation		0.000		0.177		0.000
Good	5.32 (4.15–6.50)		4.77 (3.60–5.94)		4.36 (3.27–5.45)	
Poor	10.70 (8.36–13.04)		6.15 (4.44–7.86)		10.08 (7.87–12.30)	
Mental status		0.010		0.004		0.601
Good	6.07 (4.83–7.32)		4.34 (3.27–5.40)		6.56 (5.23–7.88)	
Poor	9.16 (6.96–11.36)		7.39 (5.35–9.43)		7.20 (5.12–9.28)	

### 3.4 Multifactorial correlation analysis of delay in blood donation

The factors with significant differences in Table 2 were included in a multiple logistic regression analysis. The results showed that gender, blood donation history, sleep quality, nutritional status and mental status were independent risk factors for delay in the ALT project, with *p* < 0.05. The odds ratios (OR) and 95% confidence intervals (CI) were 5.057 (3.206–7.977), 2.735 (1.348–5.549), 1.594 (1.026–2.476), 3.679 (2.328–5.816), and 1.957 (1.243–3.080), respectively. In the BP project, age, blood donation history, sleep quality and mental state entered the final risk prediction model with *p* < 0.05. The OR and 95% CI were 0.256 (0.147–0.447), 3.658 (1.832–7.304), 6.042 (3.472–10.512), and 1.812 (1.054–3.113), respectively. In the Hb project, sex, blood donation history and nutritional status entered the final risk prediction model with p < 0.05. The OR and 95% CI were 0.244 (0.143–0.415), 0.542 (0.338–0.869), and 3.103 (2.070–4.651), respectively. Detailed data are shown in Table 3.

**Table 3:** Independent predictors of donor deferral: odds ratios and 95% confidence intervals from the multivariate logistic regression model.

Category	ALT, *n* = 2,082 (147)		PB, *n* = 2,043 (108)		Hb, *n* = 2,066 (131)	
	OR (95% CI)	*p*	OR (95% CI)	*p*	OR (95% CI)	*p*
Age		/		0.000		/
18–20	/		1		/	
21+	/		0.256 (0.147–0.447)		/	
Sex		0.000		/		0.000
Female	1		/		1	
Male	5.057 (3.206–7.977)		/		0.244 (0.143–0.415)	
Donor history		0.005		0.038		0.011
Repeat	1		1		1	
First time	2.735 (1.348–5.549)		3.658 (1.832–7.304)		0.542 (0.338–0.869)	
Sleep Quality		0.038		0.000		/
Good	1		1		/	
Poor	1.594 (1.026–2.476)		6.042 (3.472–10.512)		/	
dietary situation		0.000		/		0.000
Good	1		/		1	
Poor	3.679 (2.328–5.816)		/		3.103 (2.070–4.651)	
Mental status		0.004		0.004		/
Good	1		1		/	
Poor	1.957 (1.243–3.080)		1.812 (1.054–3.113)		/	

## 4 Discussion

The delay rate for blood donation is closely related to the screening criteria for blood donors, medical standards and local diseases in different countries. According to the World Health Organization’s 2017 global report, the global median delay rate for blood donation is 12%. There is significant variation in the delay rate among blood donors in different regions, ranging from 1 to 37% (10, 11). In this study, the delay rate of blood donation among students in Huzhou city was 12.60%, which is consistent with the median reported by the World Health Organization and lower than some Asian countries such as India (14.6%), Singapore (14.8%), and Japan (14.2%) (12–14). This difference may be due to the fact that the study population consisted mainly of students. However, Yang et al. (15), in their study conducted in a blood centre in northern China, found that the delay rate among blood donors aged 18–25 years was only 5.52% in 2017–2018, which is much lower than the results of this study. This may be due to regional differences, but there has been a gradual increase in the delay rate among the student population in recent years, which may also contribute to this difference. The study found that the delay rate among the student population has gradually increased over the last 5 years, rising from 9.37% in 2018 to 18.75% in 2022, which is a significant difference.

When analysing the reasons for blood donation delays, the study found that the majority of cases were due to temporary postponement reasons, accounting for 97.75% of the total delay. As China’s national immunisation and voluntary blood donation programmes have developed extensively, the likelihood of blood transfusion-related infections such as hepatitis B has decreased significantly among the population, especially among students who have undergone comprehensive immunisation programmes (16, 17). In addition, repeated screening during the blood donation process has led to the exclusion of a large number of donors with infectious diseases such as hepatitis B and syphilis (18), which is one of the reasons for the decreasing proportion of permanent deferrals over the years.

From the point of view of delayed items, the main reasons for delayed blood donation are failure to meet the criteria for ALT, blood pressure, and Hb. Among these, ALT has the highest delay rate, reaching 5.23%, accounting for 41.48% of all deferrals, and shows an increasing trend over the years, as shown in Figure 1. There are many reasons for elevated ALT levels, including not only pathological factors (19), but also non-pathological factors such as obesity, alcohol consumption, staying up late, fatigue and intense exercise. In this study, gender became the strongest risk factor in the risk prediction model for ALT delay, with men more likely to have elevated ALT levels than women, with an odds ratio (OR) of 5.057. Research has shown that the association between metabolic abnormalities and elevated ALT levels may vary by sex, with metabolic abnormalities being more common in young adult males (20, 21). In addition, men are more likely than women to engage in behaviours such as drinking alcohol, staying up late and exercising vigorously, which may further contribute to the development of metabolic disorders. Unhealthy diet was also identified as an independent risk factor for elevated ALT levels in this study, with an OR of 3.679.

As the Seventh Report of the Joint National Committee on Prevention, Detection, Evaluation, and Treatment of High Blood Pressure (JNC 7) lowered the threshold for diagnosis of hypertension to 140/90 mm Hg (previously 160/100 mm Hg), hypertension has gradually become a key factor in deferring blood donation (22, 23). Abnormal blood pressure (BP) was identified as the second most important reason for delayed donation in the student population in this study, with a delay rate of 3.30%, accounting for 26.22% of all deferrals, which has shown a gradual upward trend over the past 5 years. Age, blood donation history, sleep quality and mental status were identified as independent risk factors in the risk prediction model for BP delay, with OR values of 0.256, 3.658, 6.042, and 1.812, respectively. Young students, especially first-time donors, experience anxiety and fear of needles, which hinders recruitment and can lead to increased heart rate and blood pressure. This is an important reason for delayed donation (24). In modern society, there is an increasing trend for hypertension to become more prevalent in young people due to stressful lifestyles, body mass index, and habits such as alcohol consumption and smoking (25–27). In addition, the excessive use of electronic products such as the internet and smartphones in China has significantly disrupted students’ regular sleep patterns. Sleep quality plays a mediating role in the relationship between perceived stress and dietary behaviour, and between perceived stress and alcohol abuse (28). The COVID-19 pandemic may further exacerbate the general societal presence of stress, anxiety and uncertainty (29), which may be one of the reasons for the increasing trend of high BP delay rates in this study. Cao et al.’s research on the relationship between the COVID-19 pandemic and anxiety among university students found a negative relationship between social support and students’ anxiety levels. It is recommended to monitor the mental health of university students during the outbreak period (30). Therefore, the school can improve students’ mental health education, provide psychological counselling, and develop healthy sleep habits, to help them cope with stress and anxiety, strengthen their psychological resilience and emotional regulation skills, and thus enable them to better adapt to the pressures of life and learning.

In addition, low haemoglobin (Hb) levels are also an important reason for blood donation delay, with a delay rate of 2.92%, accounting for 23.15% of all deferrals. In the risk prediction model for Hb delay in this study, gender, blood donation history and nutritional status were identified as independent risk factors. Women of childbearing age who donate blood often have low haemoglobin levels due to factors such as menstrual blood loss and iron deficiency (31, 32). In addition, unhealthy eating habits driven by a desire for beauty or excessive control over diet can also lead to low haemoglobin levels in women. The results of the study showed that blood donors with good dietary habits had significantly lower delay rates compared to those with poor dietary habits. It should be noted that there was a significant increase in the Hb delay rate in 2022. Some studies have shown that before the COVID-19 pandemic, women accounted for the majority of delays due to low haemoglobin levels, whereas during the pandemic, men dominated (33). Although the results of this study still attribute the majority of Hb delays to women, further research is needed to determine whether this rapid increase is related to the COVID-19 pandemic. Schools can conduct health education courses to help students establish proper health concepts, understand nutrition science, make balanced food choices, consume sufficient nutrients, especially iron-rich foods, to maintain good physical health and reduce the risk of delayed blood donation.

It is worth noting that in the risk prediction models for the three main delay items, blood donation history was included as an independent risk factor in all final models. This finding further supports the importance of the WHO’s blood safety strategy of “collecting blood only from low-risk individuals who donate voluntarily” (9). It is a fundamental approach to ensuring blood safety in terms of quantity and quality to collectively build a stable population of “regular blood donors” within society.

## 5 Conclusion

The rate of blood donation delays in the student population is gradually increasing. Elevated ALT levels, low haemoglobin and high blood pressure are the three main factors contributing to blood donation delays among students. Non-pathological factors, poor lifestyle and emotional stress also have a significant impact on the delay rate. In order to reduce the blood donation delay rate, it is necessary to strengthen health education, increase people’s health awareness and self-management skills, especially in guiding and educating them about lifestyle choices, dietary habits and stress management. This will help to improve their understanding of self-management of health and their ability to adopt healthy behaviours before donating blood. Ultimately, this will reduce deferral rates and promote the development of a regular blood donor population.

## Data availability statement

The original contributions presented in the study are included in the article/supplementary material, further inquiries can be directed to the corresponding author.

## Ethics statement

The studies involving humans were approved by Ethics Committee of Huzhou Central Blood Station. The studies were conducted in accordance with the local legislation and institutional requirements. Written informed consent for participation was not required from the participants or the participants’ legal guardians/next of kin in accordance with the national legislation and institutional requirements.

## Author contributions

FW: Conceptualization, Formal analysis, Writing – original draft. YW: Writing – original draft. LW: Writing – review & editing. Z-gX: Writing – review & editing. H-yY: Writing – original draft. S-fW: Data curation, Methodology, Project administration, Writing – review & editing. Z-yW: Data curation, Writing – review & editing, Supervision. Y-zC: Writing – review & editing, Funding acquisition, Investigation, Resources. QS: Funding acquisition, Investigation, Writing – review & editing. J-xF: Conceptualization, Formal analysis.
